# Analysis of Clinical Indicators in Pediatric Patients with *Acinetobacter baumannii* and *Klebsiella pneumoniae* Infections

**DOI:** 10.3390/jcm15155914

**Published:** 2026-07-29

**Authors:** Yiyao Bao, Lingling Dai, Mingming Zhou, Chao Tang

**Affiliations:** National Clinical Research Center for Children and Adolescents’ Health and Diseases, Children’s Hospital, Zhejiang University School of Medicine, Hangzhou 310052, China; chbyy@zju.edu.cn (Y.B.); daill11@zju.edu.cn (L.D.); 6510053@zju.edu.cn (M.Z.)

**Keywords:** *Acinetobacter baumannii*, *Klebsiella pneumoniae*, pediatric infections, biomarkers, nosocomial infections

## Abstract

**Background:** *Acinetobacter baumannii* and *Klebsiella pneumoniae* are important causes of hospital-acquired infections in pediatric patients, but comparative data on their clinical manifestations and inflammatory biomarkers remain limited. **Methods**: We retrospectively analyzed 55 children with single-positive blood cultures for *A. baumannii* (n = 20) or *K. pneumoniae* (n = 35), together with 30 healthy controls. **Results**: CRP, IL-6, and IL-10 were higher in infected children than in healthy controls. Logistic regression identified CRP and the IL-6/IL-10 ratio as exploratory correlates of infection. In ROC analyses distinguishing infected children from healthy controls, IL-6 showed the highest AUC, followed by IL-10 and CRP. In pathogen-level analyses, the IL-6/IL-10 ratio and lipase showed exploratory discriminatory signals. **Conclusions**: In this small retrospective case–control cohort, CRP, IL-6, and IL-10 were associated with microbiologically confirmed infection. Lipase may represent a potential biomarker for differentiating *A. baumannii* from *K. pneumoniae* infection, but its clinical utility requires prospective validation in larger multicenter cohorts using clinically relevant inpatient control groups.

## 1. Introduction

Infectious diseases remain a leading global health threat to children and continue to impose a substantial burden on pediatric healthcare systems. Among these pathogens, *Acinetobacter baumannii* and *Klebsiella pneumoniae* have drawn attention because of their clinical importance and increasing prevalence among hospitalized children [1]. Both organisms can acquire and maintain antimicrobial resistance, complicating treatment and contributing to prolonged hospitalization, increased healthcare costs, and mortality [2,3]. In pediatric intensive care units, they are important causes of healthcare-associated infection [4] and can produce pneumonia, bloodstream infection, sepsis, and long-term complications [5]. The antimicrobial-resistance context also extends beyond hospitals: antimicrobial selection pressure in food-producing animals and consumer concern regarding antibiotic use in animal production form part of the wider One Health challenge [6]. The present study focuses specifically on pediatric hospital-acquired bloodstream infections and did not assess animal, food, or environmental reservoirs; therefore, no source-attribution inference is intended.

Both A. baumannii and K. pneumoniae are well-established nosocomial pathogens, but they differ in pathogenic traits and clinical behaviors that contribute to their persistence and virulence [7]. A. baumannii is particularly noted for its ability to survive in harsh environments, resist desiccation, and form biofilms on medical devices, allowing it to persist in hospital settings and cause recurrent infections that are difficult to eradicate [8]. In contrast, K. pneumoniae includes hypervirulent strains capable of causing severe invasive diseases, such as pyogenic liver abscesses and pneumonia, even in otherwise healthy hosts [9]. In pediatric patients, both pathogens can cause a wide spectrum of diseases, from respiratory tract to bloodstream infections, often associated with poor outcomes. The therapeutic challenge is further intensified by the emergence of multidrug-resistant strains that limit treatment options [10,11,12]. This dual threat of increasing virulence and antibiotic resistance underscores the need for more effective infection control programs, rapid diagnostic tools, and novel therapeutic strategies in pediatric care.

Before microbiological identification is available, *A. baumannii* and *K. pneumoniae* bloodstream infections may present with overlapping nonspecific clinical features and inflammatory abnormalities; early pathogen-level differentiation is therefore challenging. This study retrospectively analyzed children with microbiologically confirmed *A. baumannii* or *K. pneumoniae* infection and healthy controls to examine differences in routinely available clinical and inflammatory biomarkers [13,14]. The analysis focused on exploratory biomarker associations with infection status and on whether selected markers could help differentiate the two pathogens. Such biomarkers are intended to complement, rather than replace, culture-based pathogen identification. The study was not designed to evaluate clinical outcomes or to establish validated diagnostic thresholds.

## 2. Materials and Methods

### 2.1. Study Population and Design

This retrospective study was conducted following the principles of the Declaration of Helsinki and was approved by the Ethics Review Committee of the Children’s Hospital, Zhejiang University School of Medicine (Approval No.: 2025-IRB-153-P-01). Medical records of febrile pediatric patients admitted between 1 January 2020 and 31 December 2024, were systematically reviewed.

Infection groups were defined by the following inclusion criteria: (1) age < 18 years; (2) a single-positive blood culture during hospitalization confirming hospital-acquired infection caused by *A. baumannii* or *K. pneumoniae*; and (3) availability of complete clinical data. Hospital-acquired bloodstream infection was operationally defined as a clinically compatible episode with a positive blood culture obtained at least 48 h after admission and no evidence of community-acquired infection, consistent with CDC/NHSN surveillance principles.

The control group comprised healthy children with a comparable gender distribution who attended routine physical examinations during the study period. Eligibility criteria included: (1) no recent history of infection or inflammatory disease and (2) no significant past medical or family history. Healthy controls were selected to provide a noninflammatory reference group for laboratory measurements and were not intended to represent the clinical differential diagnosis among hospitalized febrile children. Accordingly, comparisons between infected and control participants should be interpreted as case-healthy-control discrimination and may overestimate diagnostic performance in clinically relevant inpatient populations.

Exclusion criteria for all groups were: (1) underlying conditions such as congenital immunodeficiency or metabolic disorders; (2) polymicrobial (mixed) infections; (3) community-acquired infections; and (4) incomplete clinical data.

**Figure 1:**
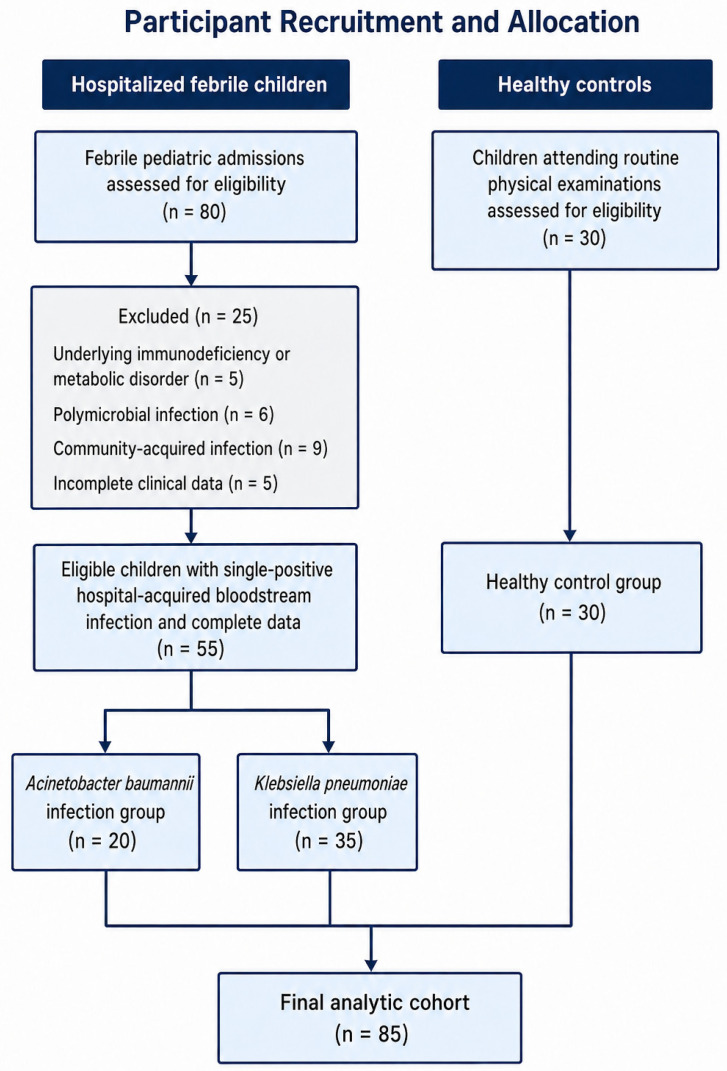
Flowchart of participant recruitment, eligibility assessment, exclusion, and final allocation of the 85 participants into the *A. baumannii* infection group (n = 20), *K. pneumoniae* infection group (n = 35), and healthy control group (n = 30).

A total of 85 participants met the eligibility requirements. They were stratified into the *A. baumannii* infection group (n = 20), the *K. pneumoniae* infection group (n = 35), and the healthy control group (n = 30). Because this was a retrospective study based on eligible records available during a fixed five-year period, the sample size was constrained by the available data rather than a prospective sample-size calculation. The modest overall and pathogen-specific sample sizes limited the number of variables that could be modeled; therefore, all regression and ROC analyses were considered exploratory. The participant-selection process is summarized in Figure 1.

### 2.2. Data Collection

Clinical data were collected from the hospital’s electronic medical record and laboratory information systems. The collected data included: (1) demographic information (age and sex); (2) blood routine indicators (white blood cell count [WBC], hemoglobin [Hb], and platelet count [PLT]); (3) inflammatory markers (CRP and cytokines including IL-2, IL-4, IL-6, IL-10, tumor necrosis factor-alpha [TNF-alpha], and interferon-gamma [IFN-gamma]); and (4) blood biochemical parameters, including liver function tests (alanine aminotransferase [ALT] and aspartate aminotransferase [AST]), electrolytes (Na^+^ and K^+^), renal function tests, and lipid profiles. Biomarker values were obtained from testing performed as part of routine clinical care. The retrospective study database did not contain complete assay-platform specifications, analytical detection limits, or intra- and inter-assay coefficients of variation for every measurement, and sampling intervals relative to blood-culture collection and antibiotic administration were not standardized; these limitations reduce reproducibility and are acknowledged in the Discussion.

### 2.3. Statistical Analysis

All statistical analyses were conducted using SPSS software, version 26.0 (IBM Corp., Armonk, NY, USA). The distribution of continuous variables was assessed for normality. Normally distributed data were presented as mean +/− standard deviation, whereas non-normally distributed data were expressed as median (interquartile range). Categorical variables were summarized as frequencies and percentages. Participants were required to have complete clinical data for inclusion; therefore, no imputation was undertaken.

For group comparisons, Student’s *t*-test or one-way analysis of variance (ANOVA) was applied to normally distributed data. The Mann–Whitney U test or Kruskal–Wallis test was used for non-normally distributed data. The chi-square test was performed to compare categorical variables. Correlations between clinical indicators were assessed using Pearson or Spearman correlation coefficients, depending on data distribution.

Binary logistic regression analysis was used to explore associations with infection status and pathogen type. Candidate variables were selected on the basis of clinical relevance and univariate findings. Owing to the limited number of observations, the models were deliberately restricted to age, sex, and two key biomarkers, which were entered simultaneously rather than selected through automated forward or backward procedures. Highly correlated cytokines with near-complete separation between groups were not entered simultaneously. Formal model-calibration assessment, goodness-of-fit reporting, and internal validation were not undertaken, and ROC-derived cutoffs were generated in the same cohort; consequently, the regression estimates and diagnostic thresholds should be regarded as exploratory and not externally validated. A two-tailed *p* value < 0.05 was considered statistically significant. Because numerous laboratory variables and pairwise subgroup contrasts were examined, the reported *p* values are unadjusted nominal values. No formal multiplicity correction was applied; therefore, isolated findings near *p* = 0.05 may represent type I error and should be interpreted as hypothesis-generating. Interpretation emphasizes the direction and magnitude of associations, confidence intervals, biological plausibility, and consistency across analyses rather than statistical significance alone.

## 3. Results

### 3.1. Univariate Analysis of Infection Characteristics

No significant differences between the two groups were observed in terms of sex, white blood cell count, pH, potassium levels, blood glucose, globulin, urea, lactate dehydrogenase, creatine kinase-MB activity, triglycerides, lipase, or interleukin (IL)-4 (*p* > 0.05). However, significant differences were observed in age, hemoglobin, platelet count, CRP, sodium, lactate, total protein, albumin, direct bilirubin, indirect bilirubin, alanine aminotransferase (ALT), aspartate aminotransferase (AST), ALT/AST ratio, cholinesterase, γ-glutamyl transferase, alkaline phosphatase, total bile acids, prealbumin, creatinine, cystatin C, creatine kinase, calcium, phosphorus, magnesium, cholesterol, IL-2, IL-6, IL-10, IL-6/IL-10 ratio, tumor necrosis factor-alpha (TNF-α), and interferon-gamma (IFN-γ) (*p* < 0.05). From a nutritional perspective, the infection group exhibited significantly lower hemoglobin, platelet count, total protein, and albumin levels compared with the control group (*p* < 0.001). Regarding liver function, ALT, AST, and total bile acid levels were significantly higher in the infection group (*p* < 0.001). From an electrolyte perspective, sodium, calcium, and phosphorus levels were significantly lower in the infection group (*p* < 0.05). For inflammatory markers, CRP, IL-6, IL-10, IL-6/IL-10 ratio, IFN-γ, and TNF-α were significantly elevated in the infection group (*p* < 0.001). Further details are presented in Table 1. These univariate comparisons were not adjusted for age. Because the control and infection groups had different age distributions and pediatric hematologic, biochemical, electrolyte, and immune reference ranges vary with age, the observed differences should be regarded as descriptive and cannot be attributed solely to infection status. In addition, the nominal *p* values were not corrected for the large number of variables examined.

### 3.2. Multivariate Logistic Regression Analysis of Infection

Binary logistic regression analysis was conducted using infection status as the dependent variable (infection group = 1; healthy control group = 0), with sex, age, CRP, and the IL-6/IL-10 ratio as prespecified independent variables. After adjustment for age and sex, CRP and the IL-6/IL-10 ratio remained statistically associated with infection, with odds ratios of 5.402 and 1.380, respectively (*p* < 0.05). IL-6 and IL-10 were not entered into the model because of near-complete separation between the infection and healthy control groups. Given the small sample and the case-healthy-control comparison, these associations should be interpreted as exploratory rather than as a validated diagnostic prediction model (Table 2). Adjustment for age and sex reduces but does not eliminate confounding, because hospitalization, acute illness severity, underlying conditions, treatment exposure, and other clinical factors were not available for comprehensive adjustment.

### 3.3. Receiver Operating Characteristic Curve Analysis of Infection Status

**Figure 2:**
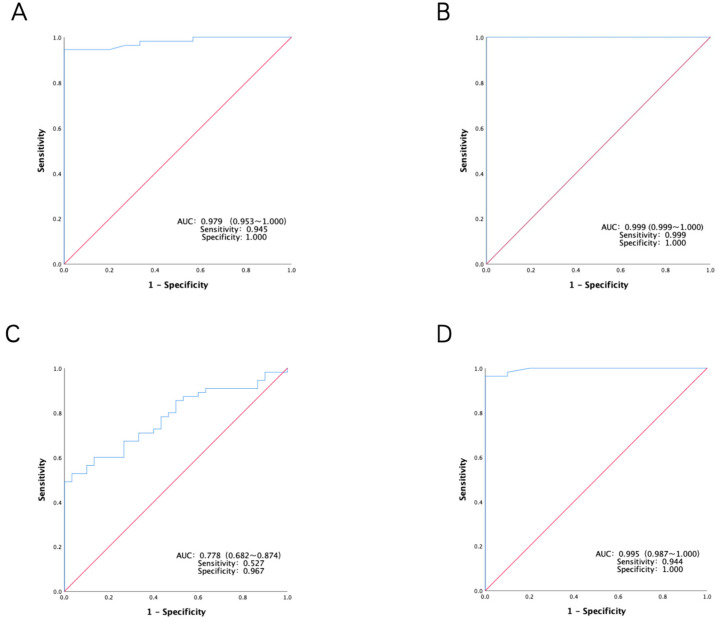
Receiver operating characteristic curves for distinguishing children with microbiologically confirmed infection (n = 55) from healthy controls (n = 30): (**A**) CRP (AUC, 0.979; 95% CI, 0.953–1.000), (**B**) IL-6 (AUC, 0.999; 95% CI, 0.999–1.000), (**C**) IL-6/IL-10 ratio (AUC, 0.778; 95% CI, 0.682–0.874), and (**D**) IL-10 (AUC, 0.995; 95% CI, 0.987–1.000). These exploratory estimates reflect case-healthy-control discrimination.

ROC curve analysis was conducted using infection status as the outcome variable (infection group = 1; healthy control group = 0), with CRP, IL-6, IL-10, and the IL-6/IL-10 ratio as test variables. Results are summarized in Figure 2 and Table 3. CRP had an AUC of 0.979 (95% CI: 0.953–1.000), IL-6 had an AUC of 0.999 (95% CI: 0.999–1.000), IL-10 had an AUC of 0.995 (95% CI: 0.987–1.000), and the IL-6/IL-10 ratio had an AUC of 0.778 (95% CI: 0.682–0.874) (all *p* < 0.001). These estimates describe discrimination between infected children and healthy controls. They should not be interpreted as evidence of equivalent diagnostic performance among hospitalized febrile children, in whom inflammatory biomarker concentrations may overlap substantially. The cutoffs were derived and evaluated in the same cohort and require external validation. In this dataset, CRP, IL-6, and IL-10 each yielded an observed specificity of 1.000; therefore, the data do not support a claim that IL-6 alone had the highest specificity. The near-perfect estimates are susceptible to spectrum bias and optimism caused by comparison with healthy children.

### 3.4. Subgroup Analysis of A. baumannii and K. pneumoniae Infections

#### 3.4.1. Univariate Analysis of Subgroup Infection Characteristics

The infection group was further divided into *A. baumannii* (n = 20) and *K. pneumoniae* (n = 35) subgroups. Univariate analysis revealed that the *K. pneumoniae* subgroup had a significantly lower age compared with the *A. baumannii* subgroup (*p* < 0.05), while sex distribution did not differ significantly (*p* > 0.05). AST levels were significantly higher in the *A. baumannii* group (*p* < 0.05), while magnesium levels were significantly lower compared with the *K. pneumoniae* group (*p* < 0.05). Notably, phosphorus levels differed between the two groups (0.92 mmol/L vs. 1.41 mmol/L, *p* < 0.05). The IL-6/IL-10 ratio and IFN-γ levels were significantly elevated in the *A. baumannii* group (*p* < 0.05). Lipase levels were markedly increased in the *A. baumannii* group but decreased in the *K. pneumoniae* group compared with the control group (*p* < 0.05). Cystatin C and urea levels were significantly higher in the *A. baumannii* group (*p* < 0.05), while creatine kinase levels were significantly reduced in the *K. pneumoniae* group (*p* < 0.001; Table 4). The marked age imbalance between the pathogen subgroups (median 42 months for the *A. baumannii* group and 7 months for the *K. pneumoniae* group) is an important source of residual confounding. Consequently, these unadjusted subgroup comparisons should be interpreted descriptively, and nominal significance across the numerous P_1_, P_2_, and P_3_ contrasts should not be regarded as evidence of pathogen-specific biological effects.

#### 3.4.2. Multivariate Logistic Regression Analysis

After adjustment for age and sex, multivariable logistic regression was conducted with pathogen type as the dependent variable (*A. baumannii* = 1; *K. pneumoniae* = 0) and the IL-6/IL-10 ratio and lipase as candidate predictors. Lipase was the only statistically significant variable (OR = 0.980, 95% CI: 0.962–0.999; *p* = 0.043) (Table 5). The odds ratio is close to 1.00, indicating a small per-unit association; this finding should therefore be interpreted cautiously and requires confirmation in larger cohorts. The estimate is based on only 20 *A. baumannii* and 35 *K. pneumoniae* cases, has an upper confidence limit close to the null, and was obtained without correction for multiple candidate comparisons. The borderline nominal *p* value (0.043) may be sensitive to influential observations; individual-level outlier and stability analyses were not available. Accordingly, this result does not establish lipase as a validated pathogen-specific biomarker.

#### 3.4.3. ROC Curve Analysis for Discriminating Pathogens

**Figure 3:**
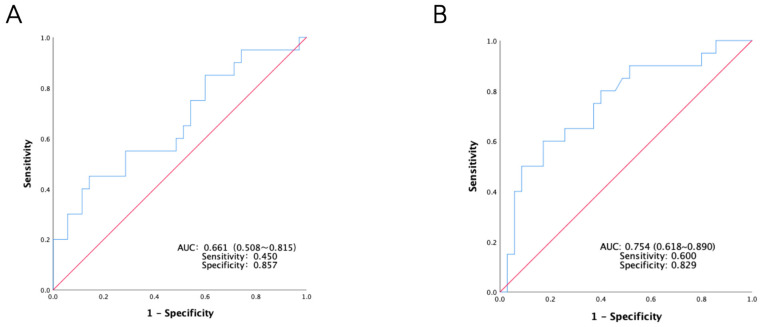
Receiver operating characteristic curves for differentiating *A. baumannii* infection (n = 20) from *K. pneumoniae* infection (n = 35): (**A**) IL-6/IL-10 ratio (AUC, 0.661; 95% CI, 0.508–0.815) and (**B**) lipase (AUC, 0.754; 95% CI, 0.618–0.890). The estimates were derived and evaluated in the same small retrospective cohort and should be interpreted as exploratory.

ROC curve analysis was conducted with pathogen type as the state variable (*A. baumannii* = 1; *K. pneumoniae* = 0) using the IL-6/IL-10 ratio and lipase as test variables. Results are summarized in Figure 3 and Table 6. The IL-6/IL-10 ratio had an AUC of 0.661 (95% CI: 0.508–0.815), with sensitivity of 0.450 and specificity of 0.857. Lipase had an AUC of 0.754 (95% CI: 0.618–0.890), with sensitivity of 0.600 and specificity of 0.829. Thus, lipase showed only moderate discriminatory performance and should be regarded as a hypothesis-generating candidate biomarker rather than an established diagnostic marker.

## 4. Discussion

This retrospective study describes clinical and laboratory differences among children with bloodstream infection caused by *Acinetobacter baumannii* or *Klebsiella pneumoniae* and healthy controls. The lower hemoglobin, platelet, total-protein, and albumin values and the differences in hepatic, renal, electrolyte, and inflammatory indices observed in infected children are compatible with the systemic effects of acute illness. However, these findings cannot be attributed specifically to either pathogen, because the comparison simultaneously captures differences in age, hospitalization, acute inflammatory status, illness severity, underlying disease, nutritional condition, and treatment exposure. The cross-sectional retrospective data also do not establish that inflammation caused nutritional depletion or organ dysfunction. These laboratory differences should therefore be interpreted as descriptive associations that require confirmation in age-matched hospitalized comparator groups.

In this case–healthy-control comparison, CRP, IL-6, and IL-10 were substantially higher in infected children. Previous pediatric studies likewise found that IL-6 and CRP were higher in children with bacteremia than in children with less severe infections, although the reported discriminatory performance was more modest when clinically infected comparator groups were used [15]. In pediatric hematology–oncology populations, IL-6 and IL-10 have also been associated with bacteremia and severe infection [16]. Studies of pediatric sepsis further suggest that IL-6, IL-10, and CRP may help distinguish bacterial phenotypes, but their performance is influenced by disease severity, infection site, and the comparator population [17,18]. Accordingly, the near-perfect AUCs observed here are likely to reflect, at least in part, the contrast between hospitalized infected children and healthy controls and should not be generalized to routine inpatient diagnostic triage. CRP, IL-6, and IL-10 are nonspecific host-response markers, and the present study does not demonstrate that any of them can identify *A. baumannii* or *K. pneumoniae* before microbiological confirmation.

The exploratory pathogen-level analysis identified a difference in lipase between the *Acinetobacter baumannii* and *Klebsiella pneumoniae* groups. Lipase had an AUC of 0.754, sensitivity of 0.600, and specificity of 0.829, while the adjusted odds ratio was 0.980 per unit (95% CI, 0.962–0.999; nominal *p* = 0.043). These estimates indicate moderate discrimination and a small per-unit association, not evidence of a clinically established biomarker. The small subgroup sizes, wide dispersion of lipase values, lack of multiplicity correction, and potential influence of outliers further limit robustness. No mechanistic inference can be made because pancreatic imaging, amylase measurements, documented pancreatitis, serial sampling, and experimental assessments of pancreatic injury were unavailable. Lipase should therefore be considered only a hypothesis-generating candidate for prospective evaluation in larger, age-matched, multicenter cohorts.

This study has several limitations. First, its retrospective single-center design and modest sample size, particularly the 20 *A. baumannii* and 35 *K. pneumoniae* cases, limit statistical power and increase the risk of unstable regression and ROC estimates. Second, healthy children were used as the reference group. This design introduces spectrum bias and is likely to overestimate discrimination relative to the clinically relevant setting of hospitalized febrile children with culture-negative sepsis, other infections, or noninfectious inflammatory conditions. Third, age was substantially imbalanced across the control and pathogen groups. Although age and sex were included in the restricted regression models, residual age confounding remains likely because pediatric hematologic, biochemical, electrolyte, and cytokine values change with development. Fourth, numerous laboratory variables and three families of subgroup contrasts were examined without formal multiple-testing correction. The reported *p* values are therefore nominal, and findings near 0.05 may represent type I error. Fifth, the multivariable models could include only a small number of predictors and were not internally or externally validated. Important potential confounders, including illness severity, ICU admission, mechanical ventilation, septic shock, underlying disease, prior antimicrobial exposure, nutritional status, and duration of hospitalization, were unavailable for comprehensive adjustment. Sixth, antimicrobial susceptibility profiles, including carbapenem resistance and extended-spectrum beta-lactamase production, and clinically important outcomes such as mortality, organ dysfunction, ICU stay, and total length of hospitalization were not analyzed. Seventh, assay-platform specifications, analytical detection limits, coefficients of variation, and standardized sampling times relative to blood culture and antibiotic administration were incompletely available. Finally, the cut-offs were derived and evaluated in the same cohort, and local patient characteristics and clinical practices further limit generalizability. Future prospective multicenter studies should recruit age-matched hospitalized comparator groups, prespecify a limited biomarker panel, apply multiplicity control, standardize assays and sampling, collect resistance and outcome data, and perform internal and external validation before clinical use is considered.

## 5. Conclusions

In this small retrospective single-center case–control study, CRP, IL-6, and IL-10 were associated with microbiologically confirmed *A. baumannii* or *K. pneumoniae* infection when compared with healthy controls. These findings should not be interpreted as validated diagnostic performance in hospitalized febrile children. Lipase showed moderate exploratory ability to differentiate *A. baumannii* from *K. pneumoniae* infection and should be considered a potential biomarker that warrants prospective validation. Larger multicenter studies incorporating clinically relevant controls, standardized assays, antimicrobial susceptibility data, clinical outcomes, and external validation are needed before these markers can inform routine clinical decision-making. The reported cut-offs should not be used for clinical decision-making or pathogen-directed treatment in the absence of prospective validation.

## Figures and Tables

**Table 1:** Clinical characteristics of the patient groups.

	Control Group (n = 30)	Infection Group (n = 55)	*p*-Value
Age (months)	54.50 (7.75, 117.00)	17.00 (3.00, 84.00)	0.038
Sex (male/female)	10/20	30/25	0.727
WBC (×10^9^/L)	6.12 (5.19, 7.45)	4.57 (0.34, 10.43)	0.493
HB (g/L)	132.50 (124.00, 143.00)	91.00 (75.00, 105.00)	<0.001
PLT (×10^9^/L)	307.50 (249.50, 349.50)	103.00 (15.00, 260.00)	<0.001
CRP (mg/dL)	1.75 (0.95, 2.30)	56.47 (27.53, 80.95)	<0.001
PH	7.37 (7.35, 7.39)	7.40 (7.34, 7.44)	0.092
K^+^ (mmol/L)	3.90 (3.70, 4.02)	3.90 (3.60, 4.20)	0.890
Na^+^ (mmol/L)	138.50 (137.00, 140.00)	135.00 (132.00, 138.00)	<0.001
Glu (mmol/L)	5.30 (5.00, 5.80)	5.50 (4.90, 6.40)	0.699
Lac (mmol/L)	1.60 (1.30, 1.90)	2.10 (1.50, 2.60)	0.003
Total protein (g/L)	66.30 (63.12, 69.43)	54.80 (50.70, 60.90)	<0.001
Albumin (g/L)	44.10 (42.90, 47.75)	33.90 (30.80, 38.40)	<0.001
Globulin (g/L)	21.95 (18.58, 24.73)	20.50 (15.50, 23.70)	0.111
Direct bilirubin (μmol/L)	1.80 (1.35, 2.32)	3.80 (1.70, 25.30)	<0.001
Indirect bilirubin (μmol/L)	6.55 (4.22, 9.92)	9.70 (4.80, 24.00)	0.039
ALT (U/L)	14.50 (11.00, 17.25)	29.00 (19.00, 81.00)	<0.001
AST (U/L)	25.00 (20.00, 29.50)	49.00 (33.00, 77.00)	<0.001
AST/ALT	1.67 (1.39, 2.12)	1.48 (0.85, 1.83)	0.039
Cholinesterase (U/L)	8709.50 (8235.00, 10044.00)	5243.00 (3613.00, 6636.00)	<0.001
γ -glutamyl transferase (U/L)	14.50 (12.00, 18.25)	50.00 (23.00, 108.00)	<0.001
Alkaline phosphatase (U/L)	266.50 (171.75, 354.00)	186.00 (102.00, 263.00)	0.010
Total bile acid (μmol/L)	2.85 (1.60, 6.05)	7.20 (3.50, 21.70)	<0.001
Prealbumin (mg/L)	233.70 (212.08, 266.95)	131.60 (92.10, 175.70)	<0.001
Creatinine (μmol/L)	38.50 (28.75, 51.25)	26.00 (18.00, 38.00)	<0.001
Urea (mmol/L)	4.56 (3.62, 5.56)	4.11 (3.23, 6.58)	0.682
Cystatin C (mg/L)	0.72 (0.66, 0.82)	0.86 (0.68, 1.22)	0.013
Lactate dehydrogenase (U/L)	215.50 (173.00, 308.00)	286.00 (187.00, 374.00)	0.103
Creatine kinase (U/L)	75.00 (63.75, 114.75)	32.00 (21.00, 71.00)	<0.001
Creatine kinase-MB activity (U/L)	17.50 (14.75, 23.25)	20.00 (13.00, 30.00)	0.723
Calcium (mmol/L)	2.41 (2.35, 2.51)	2.18 (2.03, 2.30)	<0.001
Phosphorus (mmol/L)	1.58 (1.45, 1.70)	1.27 (0.83, 1.55)	0.001
Magnesium (mmol/L)	0.81 (0.75, 0.85)	0.74 (0.66, 0.82)	0.009
Triglyceride (mmol/L)	1.01 (0.85, 1.47)	1.10 (0.73, 1.55)	0.982
Cholesterol (mmol/L)	4.26 (3.50, 4.81)	3.10 (2.45, 4.10)	<0.001
Lipase (U/L)	14.40 (9.25, 19.60)	12.30 (7.10, 26.00)	0.502
IL-2 (pg/mL)	4.65 (3.65, 5.45)	3.10 (2.10, 4.70)	0.001
IL-4 (pg/mL)	2.75 (2.28, 3.20)	2.50 (1.90, 3.00)	0.250
IL-6 (pg/mL)	5.45 (3.80, 12.60)	1499.40 (150.90, 6432.00)	<0.001
IL-10 (pg/mL)	3.40 (2.80, 4.30)	146.20 (24.30, 1151.40)	<0.001
IL-6/IL-10	1.50 (1.05, 3.88)	5.12 (1.98, 19.39)	<0.001
TNF (pg/mL)	2.25 (1.28, 3.35)	3.20 (1.90, 9.70)	0.012
IFN-γ (pg/mL)	3.50 (2.38, 3.62)	6.50 (3.80, 19.50)	<0.001

Values are expressed as median (interquartile range). Continuous variables are reported to two decimal places when applicable; *p* values are reported to three decimal places, with values < 0.001 shown as <0.001. Abbreviations: WBC, white blood cell count; HB, hemoglobin; PLT, platelet count; CRP, C-reactive protein; ALT, alanine aminotransferase; AST, aspartate aminotransferase; IL, interleukin; TNF, tumor necrosis factor; IFN, interferon. All *p* values in Table 1 are unadjusted nominal values. Because multiple laboratory variables were tested, the table is exploratory and individual *p* values, particularly those close to 0.05, should not be interpreted as confirmatory evidence.

**Table 2:** Logistic regression of key indicators (CRP and IL-6/IL-10).

	b	OR	95% CI	*p*-Value
CRP	1.687	5.402	1.635–17.846	0.006
IL-6/IL-10	0.322	1.380	1.086–1.755	0.009

Abbreviations: OR, odds ratio; CI, confidence interval; CRP, C-reactive protein; IL, interleukin.

**Table 3:** Exploratory ROC analysis distinguishing hospitalized children with microbiologically confirmed infection from healthy controls.

	Cut-Off Value	AUC	95% CI	Sensitivity	Specificity	*p*-Value
CRP	3.78	0.979	0.953–1.000	0.945	1.000	<0.001
IL-6	22.95	0.999	0.999–1.000	0.999	1.000	<0.001
IL-10	5.60	0.995	0.987–1.000	0.944	1.000	<0.001
IL-6/IL-10	4.98	0.778	0.682–0.874	0.527	0.967	<0.001

Abbreviations: AUC, area under the curve; CI, confidence interval; CRP, C-reactive protein; IL, interleukin. The cut-off values in this table were optimized for infected children versus healthy controls. They must not be transferred to pathogen-level discrimination or routine inpatient triage.

**Table 4:** Analysis of infection subgroups.

	Control Group (n = 30)	*Acinetobacter baumannii* Group (n = 20)	P_1_	*Klebsiella pneumoniae* Group (n = 35)	P_2_	P_3_
Age (months)	54.50 (7.75, 117.00)	42 (16.5, 105.5)	0.992	7 (3, 36)	0.003	0.001
Sex (male/female)	10/20	10/10	0.239	20/15	0.055	0.609
WBC (×10^9^/L)	6.12 (5.19, 7.45)	3.525 (0.175, 9.445)	0.122	6.12 (0.46, 12.64)	0.963	0.151
HB (g/L)	132.5 (124, 143)	88 (81.25, 101.5)	<0.001	93 (71, 106)	<0.001	0.779
PLT (×10^9^/L)	307.5 (249.5, 349.5)	34 (15.25, 216.5)	<0.001	112 (14, 288)	<0.001	0.192
CRP (mg/dL)	1.75 (0.95, 2.3)	57.165 (14.55, 114.6475)	<0.001	55.21 (29.19, 76.84)	<0.001	0.875
PH	7.3655 (7.3458, 7.3910)	7.4025 (7.3448, 7.4513)	0.16	7.391 (7.342, 7.427)	0.146	0.588
K^+^ (mmol/L)	3.9 (3.7, 4.02)	3.6 (3.25, 4.075)	0.1	4 (3.7, 4.4)	0.197	0.018
Na^+^ (mmol/L)	138.5 (137, 140)	135 (131.25, 138.75)	0.006	135 (132,138)	0.001	0.895
Glu (mmol/L)	5.3 (5, 5.8)	5.45 (4.95, 7.275)	0.565	5.5 (4.7, 6.4)	0.864	0.779
Lac (mmol/L)	1.6 (1.3, 1.9)	1.95 (1.275, 2.3)	0.106	2.4 (1.5, 2.7)	0.001	0.175
Total protein (g/L)	66.3 (63.12, 69.43)	55 (52.175, 60.9)	<0.001	53.9 (45.5, 60.8)	<0.001	0.793
Albumin (g/L)	44.1 (42.9, 47.75)	33.15 (29.1, 37.875)	<0.001	34.9 (31.1, 38.6)	<0.001	0.294
Globulin (g/L)	21.95 (18.58, 24.73)	21.6 (18.625, 24.3)	0.976	19.2 (14, 22.1)	0.024	0.096
Direct bilirubin (μmol/L)	1.8 (1.35, 2.32)	3.55 (1.2, 32.075)	0.053	3.9 (1.9, 15.4)	<0.001	0.753
Indirect bilirubin (μmol/L)	6.55 (4.22, 9.92)	8.25 (3.575, 22.625)	0.303	10.4 (5.4, 25.1)	0.024	0.426
ALT (U/L)	14.5 (11, 17.25)	40.5 (20, 87)	0.001	27 (19, 81)	<0.001	0.753
AST (U/L)	25 (20, 29.5)	60 (40.75, 106)	<0.001	38 (27, 69)	<0.001	0.025
AST/ALT	1.6711 (1.3948, 2.1237)	1.6368 (0.9671, 2.7157)	0.905	1.2222 (0.6897, 1.7)	0.004	0.072
Cholinesterase (U/L)	8709.5 (8235, 10044)	5390.5 (3126.25, 6572.75)	<0.001	5243 (3705, 6830)	<0.001	0.501
γ-glutamyl transferase (U/L)	14.5 (12, 18.25)	46.5 (24.25, 81.25)	<0.001	53 (23,123)	<0.001	0.779
Alkaline phosphatase (U/L)	266.5 (171.75, 354)	123.5 (78.25, 277.25)	0.007	210 (124, 263)	0.06	0.107
Total bile acid (μmol/L)	2.85 (1.6, 6.05)	7.65 (3.8, 27.45)	0.001	6.2 (3.4, 19.9)	0.001	0.462
Prealbumin (mg/L)	233.7 (212.08, 266.95)	138.5 (112.175, 174.7)	<0.001	124.3 (84.8, 178.3)	<0.001	0.391
Creatinine (μmol/L)	38.5 (28.75, 51.25)	27 (23.25, 41.75)	0.022	25 (17, 35)	<0.001	0.244
Urea (mmol/L)	4.56 (3.62, 5.56)	5.365 (3.745, 11.0825)	0.259	3.71 (3.16, 5.8)	0.182	0.025
Cystatin C (mg/L)	0.718 (0.659, 0.816)	0.955 (0.684, 1.337)	0.036	0.84 (0.665, 1.167)	0.037	0.668
Lactate dehydrogenase (U/L)	215.5 (173, 308)	310.5 (184, 493)	0.08	281 (187, 321)	0.242	0.259
Creatine kinase (U/L)	75 (63.75, 114.75)	31.5 (19.5, 94.5)	0.002	32 (21, 71)	<0.001	0.972
Creatine kinase-MB activity (U/L)	17.5 (14.75, 23.25)	16 (11, 29.5)	0.506	22 (13.4, 30)	0.343	0.293
Calcium (mmol/L)	2.41 (2.35, 2.51)	2.165 (1.935, 2.25)	<0.001	2.23 (2.04, 2.37)	<0.001	0.175
Phosphorus (mmol/L)	1.585 (1.4525, 1.7)	0.92 (0.6725, 1.41)	<0.001	1.41 (1.11, 1.77)	0.055	0.01
Magnesium (mmol/L)	0.81 (0.75, 0.85)	0.715 (0.5125, 0.77)	0.002	0.78 (0.68, 0.85)	0.101	0.023
Triglyceride (mmol/L)	1.01 (0.85, 1.47)	1.565 (0.8325, 1.9025)	0.109	0.93 (0.6, 1.39)	0.272	0.009
Cholesterol (mmol/L)	4.265 (3.5, 4.81)	3.4 (2.2475, 3.8025)	0.005	3.06 (2.55, 4.12)	<0.001	0.903
Lipase (U/L)	14.4 (9.25, 19.6)	23.5 (11.775, 42.825)	0.035	9.4 (6.5, 15.8)	0.018	0.002
IL-2 (pg/mL)	4.65 (3.65, 5.45)	3.05 (1.775, 5)	0.017	3.1 (2.1, 4.4)	0.002	0.986
IL-4 (pg/mL)	2.75 (2.28, 3.20)	2.4 (1.8, 3.3)	0.275	2.5 (2.1, 2.9)	0.356	0.569
IL-6 (pg/mL)	5.45 (3.80, 12.60)	1548.9 (191.35, 6257.05)	<0.001	1499.4 (129.3, 6918.2)	<0.001	0.662
IL-10 (pg/mL)	3.4 (2.8, 4.3)	90.7 (15.575, 739.8)	<0.001	195.9 (65.3, 1182.7)	<0.001	0.214
IL-6/IL-10	1.5 (1.05, 3.88)	8.27 (3.46, 41.59)	<0.001	4.82 (1.57, 9.17)	0.002	0.042
TNF (pg/mL)	2.25 (1.28, 3.35)	3.2 (1.9, 28.125)	0.042	3.2 (1.6, 9.7)	0.024	0.827
IFN-γ (pg/mL)	3.5 (2.38, 3.62)	5.2 (3.2, 21.525)	0.01	7 (4, 19.5)	<0.001	0.546

P_1_: Control group vs. *Acinetobacter baumannii* group. P_2_: Control group vs. *Klebsiella pneumoniae* group. P_3_: *Acinetobacter baumannii* group vs. *Klebsiella pneumoniae* group. Values are expressed as median (interquartile range). Abbreviations: WBC, white blood count; HB, hemoglobin; PLT, platelet; CRP, C-reactive protein; ALT, alanine transaminase; AST, aspartate aminotransferase; IL, interleukin; TNF, tumor necrosis factor; IFN, interferon. P_1_, P_2_, and P_3_ are unadjusted nominal *p* values. No multiplicity correction was applied across the three contrast families and numerous laboratory variables; therefore, Table 4 is exploratory and findings close to *p* = 0.05 require independent confirmation.

**Table 5:** Logistic regression of key indicators.

	b	OR	95% CI	*p*-Value
IL-6/IL-10	−0.028	0.972	0.939–1.007	0.115
Lipase	−0.200	0.980	0.962–0.999	0.043

Abbreviations: OR, odds ratio; CI, confidence interval; IL, interleukin. Estimates are exploratory, adjusted for age and sex, and derived from a small single-center subgroup sample. The reported *p* values are nominal and were not adjusted for multiple testing.

**Table 6:** ROC curve analysis for differentiating *Acinetobacter baumannii* and *Klebsiella pneumoniae*.

	Cut-Off Value	AUC	95% CI	Sensitivity	Specificity	*p*-Value
IL-6/IL-10	17.99	0.661	0.508–0.815	0.450	0.857	0.048
Lipase	19.70	0.754	0.618–0.890	0.600	0.829	0.002

Abbreviations: AUC, area under the curve; CI, confidence interval; IL, interleukin. The IL-6/IL-10 cut-off of 17.99 was optimized specifically for differentiating *A. baumannii* from *K. pneumoniae* in this cohort. It differs from the cut-off of 4.98 in Table 3 because Table 3 classifies infected children versus healthy controls. Neither threshold has undergone internal or external validation.

## Data Availability

All data generated or analyzed during this study are included in this article. Further inquiries can be directed to the corresponding author.

## Abbreviations

The following abbreviations are used in this manuscript:

WBC	White blood count
HB	Hemoglobin
PLT	Platelet
CRP	C-reactive protein
ALT	Alanine transaminase
AST	Aspartate aminotransferase
IL	Interleukin
TNF	Tumor necrosis factor
IFN	Interferon
